# La Soufrière volcanic eruption in 2021 was not responsible for the high Fe, Al, or Mn concentrations found in stranded *Sargassum* spp.

**DOI:** 10.1073/pnas.2411350121

**Published:** 2024-08-08

**Authors:** Tristan Gobert, Solène Connan, Benjamin Châtelain, Marie-Laure Rouget, Valérie Stiger-Pouvreau, Matthieu Waeles

**Affiliations:** ^a^University of Brest, CNRS, Institut de recherche pour le développement, Ifremer, Laboratoire des sciences de l'environnement marin, Plouzane F-29280, France; ^b^University of Brest, Pôle Spectrométrie Océan, Unité mixte de service 3113, Institut Universitaire Européen de la Mer, Plouzane F-29280, France

Machado et al. (1) observed from August 2021 increases in Fe, Al, and Mn content in holopelagic *Sargassum* spp. stranded in Jamaica. They attributed these increases to a probable interaction that took place 3 to 4 mo earlier, between the metal-rich volcanic ash deposited in the oceanic surface layer following the eruption of La Soufrière in April 2021 and the *Sargassum* spp. rafts when they were east of St Vincent. We demonstrate here that i) *Sargassum* spp., collected along this route to Jamaica, i.e., in June 2021 near the French Caribbean Islands (FCI), did not present any Fe, Al, or Mn anomalies and that ii) the general composition of stranded biomass, with high Fe, Al, or Mn levels as observed by Machado et al. (1), is acquired during the transit of algae rafts in the coastal zone.

**Fig. 1.**
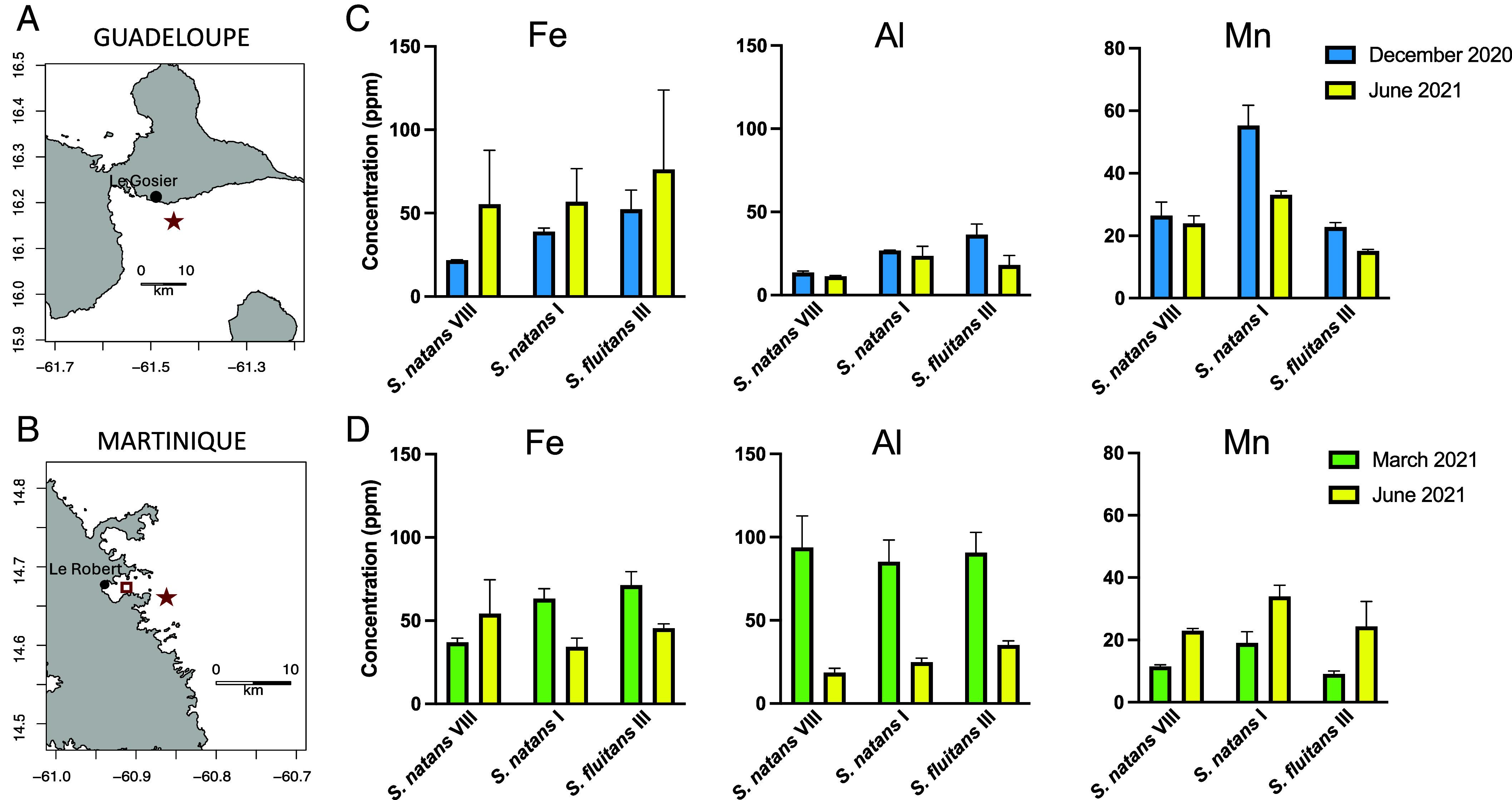
Maps of (*A*) Guadeloupe and (*B*) Martinique east coasts were *Sargassum* spp. samples were collected before and after La soufrière eruption at stations located 3 to 4 km offshore (red stars). Samples off Guadeloupe were collected on 7 December 2020 and 10 June 2021 (60 d after the eruption). Samples off Martinique were collected on 9 March 2021 and 15 June 2021 (65 d after the eruption); (*C*) Fe, Al, and Mn concentrations in the three *Sargassum* spp. morphotypes for samples collected off Guadeloupe; (*D*) Fe, Al, and Mn concentrations in the three *Sargassum* spp. morphotypes for samples collected off Martinique. Analyses of samples were conducted following the method described in ref. 2. Each morphotype was sampled in triplicate. Values are mean ± SD (*n* = 3). All data are reported in ppm dry weight (ppm).

We collected *Sargassum* spp. 3 to 4 km off Guadeloupe and Martinique (FCI) in December 2020, March 2021, and June 2021 (Fig. 1). According to estimates by Machado et al. (1), based on sargasso raft trajectories, the *Sargassum* spp. we collected off FCI (61°W) in June 2021 (~60 d after the eruption) came directly from the area east of St. Vincent down to 50°W where they shared the sea surface for ~50 d with volcanic ashes. Our analyses show that all *Sargassum* spp. morphotypes off Guadeloupe and Martinique contained Fe and Al at levels below 100 ppm and Mn at levels below 60 ppm. Such “low” levels in offshore biomass are consistent with those reported in previous years over the western tropical North Atlantic Ocean (2, 3) and do not differ with the levels found off Guadeloupe and off Martinique over the months preceding La Soufrière eruption (Fig.1).

**Fig. 2.**
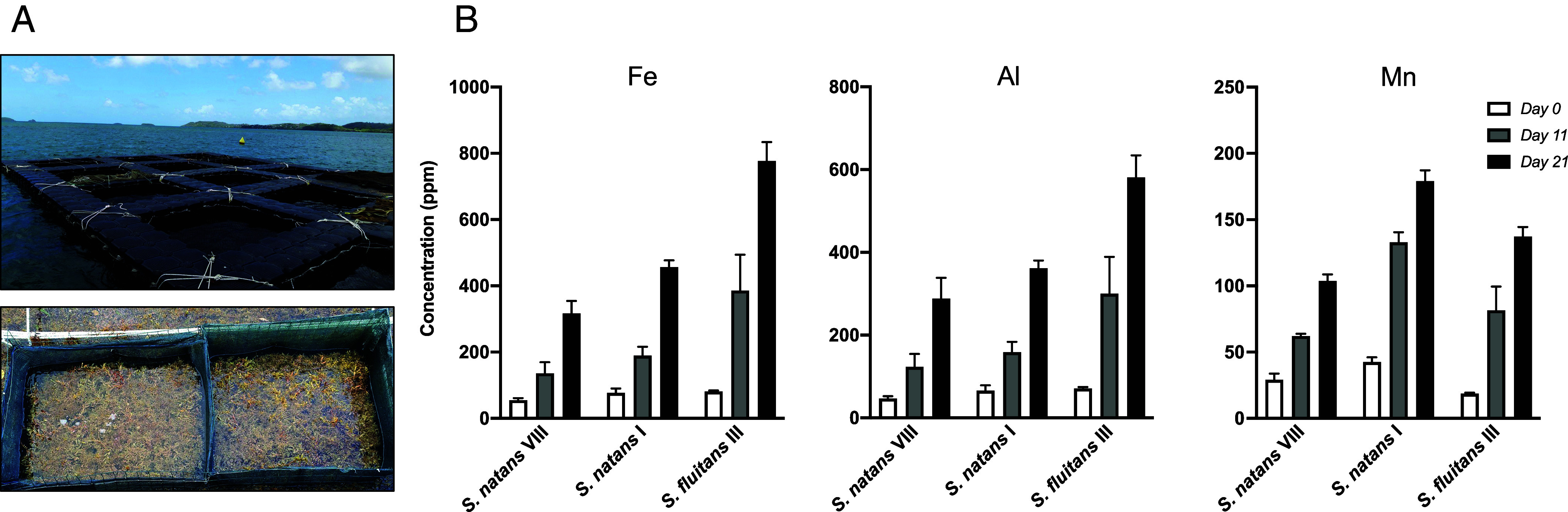
(*A*) Photos of the 21 d “cage” experiment conducted from 26 July to 16 August 2021 in Baie du Robert on the east coast of Martinique (the red square in Fig. 1*B* shows the location of the experimental site). For this experiment, *Sargassum* spp. biomass was harvested at the offshore site (red star in Fig. 1*B*), then transported by boat to Baie du Robert and placed in three different cages at a density of 3 kg m^−3^ (1 kg of each morphotype per cage). Over the 21-d experiment, the *Sargassum* spp. maintained a constant photosynthetic activity in all cages (F_v_/F_m_ = 0.8 ± 0.1). (*B*) Evolution of Fe, Al, and Mn concentrations (ppm) in the three *Sargassum* spp. morphotypes over the cage experiment. Samples of each morphotype were taken from three different cages on days 0, 11, and 21. Values are mean ± SD (*n* = 3).

The “high” metal levels observed by Machado et al. (1) over their 2021 survey in Jamaica (e.g., range 200 to 800 ppm for Fe and Al) are not unusual for stranded biomass, as reported for example in Dominican Republic in 2015 (4), in Martinique in 2017 (2) or in Jamaica in 2019 (5). These high levels, which can be an order of magnitude above open-ocean levels, can only be explained by rapid changes in composition over the transit of *Sargassum* spp. through the coastal zone where they interact with waters richer in terrigenous elements as compared to open-ocean waters. We managed to reproduce and quantify these changes through an experiment in which we monitored a biomass collected off Martinique and then transferred in a cage for 3 wk in Baie du Robert (Fig. 2). The results clearly show that all three morphotypes of *Sargassum* spp. considerably enriched, with levels doubling or tripling after 11 d and still rising sharply up to 21 d. Following studies will need to address how contaminants such as As and Cd are affected by these major changes in the coastal zone.
